# Affective Dependence and Aggression: An Exploratory Study

**DOI:** 10.1155/2014/805469

**Published:** 2014-06-23

**Authors:** Filippo Petruccelli, Pierluigi Diotaiuti, Valeria Verrastro, Irene Petruccelli, Roberta Federico, Giovanni Martinotti, Andrea Fossati, Massimo Di Giannantonio, Luigi Janiri

**Affiliations:** ^1^Department of Human, Social and Health Sciences, University of Cassino, 03043 Cassino, Italy; ^2^Faculty of Human and Social Sciences, Kore University of Enna, 94100 Enna, Italy; ^3^Department of Neuroscience and Imaging, Institute of Psychiatry, G. d'Annunzio University of Chieti-Pescara, 66100 Chieti, Italy; ^4^Department of Human Sciences, Lumsa University of Rome, 00193 Rome, Italy; ^5^Institute of Psychiatry, Catholic University of Rome, 00198 Rome, Italy

## Abstract

*Introduction.* Emotionally dependent subjects may engage in controlling, restrictive, and aggressive behaviours, which limit their partner's autonomy. The underlying causes of such behaviours are not solely based on levels of aggression, but act as a mean of maintaining the subject's own sense of self-worth, identity, and general functioning. *Objective.* The aim of the paper is to explore the correlation between affective dependency and reactive/proactive aggression and to evaluate individual differences as predisposing factors for aggressive behaviour and emotional dependency. *Methods.* The *Spouse-Specific Dependency Scale* (SSDS) and the *Reactive Proactive Questionnaire* (RPQ) were administered to a sample of 3375 subjects. *Results.* In the whole sample, a positive correlation between emotional dependency and proactive aggression was identified. Differences with regard to sex, age group, and geographical distribution were evidenced for the scores of the different scales. *Conclusion*. A fundamental distinction between reactive and proactive aggression was observed, anchoring proactive aggression more strictly to emotional dependency. Sociocultural and demographical variables, together with the previous structuring of attachment styles, help to determine the scope, frequency, and intensity of the demands made to the partner, as well as to feed the fears of loss, abandonment, or betrayal.

## 1. Introduction

Researchers are increasingly recognizing emotional dependency as a primary precursor of violence within relationships [1]. Murphy et al. [2] found that physically violent men show extremely high levels of emotional dependency in comparison with subjects in both happy and unhappy relationships and therefore concluded that emotionally dependent individuals are more likely to resort to violence than not emotionally dependent ones, also if in an unhappy relationship. Physically violent men have lower self-esteem and higher abandonment fears, even if compared to subjects in unhappy relationships [2]. Men who use violence against their partners usually focus solely on the affair, excluding many other social contacts. Other studies have been undertaken in order to compare levels of aggression and emotional security, aiming to assess whether emotional dependence presents a greater risk for aggression in domestic violence incidents [1]. A research evidenced that violent men have higher levels of aggression, but also higher levels of emotional insecurity. Therefore, high levels of emotional dependency rather than high levels of aggression have been interpreted as a significant precursor of physical violence. Violence and controlling behaviors may result in abusive relationships; many researchers have shown that the underlying causes of those behaviors are not only based on levels of aggression but also acted as a mean of maintaining the subject's own sense of self-worth, identity, and general functioning [3]. A higher level of dependence may generate attachment anxieties, manifested through higher levels of intimate jealousy, more impaired intimate interpersonal competence, and increased perpetration and severity of perpetrated intimate aggression. Among male batterers, maladaptive interpersonal dependence has been conceptualized as a consequence of childhood insecure attachment and a mean to preserve the male ego [4]. Kinsfogel and Grych [5] noted that the characteristics of emotional dependency, high need for intimacy, and abandonment anxiety are consequences of a preoccupied attachment style. According to the attachment theory, the attachment system is not limited to childhood and extends to emotional relationships (e.g., friendships, marital relationships, etc.). Attachment styles are usually resistant to modification and change [6]. Recent studies [7] confirmed a positive and significant correlation between obsessive love and ambivalent attachment style. These results are congruent with the studies of Feeney and Noller [8], Hamidi [9], and Arefi et al. [10]. Two types of dependence have been traditionally considered: instrumental and emotional. The first one is characterized by insecurity, lack of autonomy in daily life and lack of initiative, seeking for social support, helplessness, and difficulties in making decisions, in taking responsibility and in developing with efficiency. Emotional dependence is instead characterized by excessive emotional demands, narrow interpersonal relationships, unbalanced relationships (in which submission and idealization of the partner prevails), low self-esteem, and urgent need of the other, leading to an excessively clinging behaviour and an intense fear of loneliness. Research has paid particular attention to the relation between interpersonal dependence, domestic violence, and marital conflict [11, 12], finding that female victims of domestic violence show high dependence on their partners [13, 14]. Excessive dependency may be a factor that increases tolerance on abuses acted by the partner and may hinder the end of an abusive relationship [15, 16]. On the other hand, many studies have shown that male abusers are more dependent on their partners in comparison with men who are not violent in romantic relationships [17]. Although in most cases the violent behaviour occurs at the hands of the male subject, our analysis will focus on detecting the types and levels of emotional dependency in both the elements of the couple; in fact, codependency and intersection between specific types of dependence may increase the probability of the occurrence of aggressions. Further qualitative distinctions are to be taken into account in analysing the levels of dependency of the members of a couple: aggressive behaviors may be defined primarily as being reactive or proactive [18–20]. Several researches have suggested that individuals displaying reactive aggression may be differentiated from individuals displaying proactive aggression by measures of personality and psychopathology, as well as history of aggression, and type and severity of aggressive behaviors committed [21]. The distinction between proactive and reactive aggression represents a potentially relevant perspective that may shed light on different etiological pathways to aggression [18, 22]. Classifications typically identify two subtypes of aggression: the first is characterized by planning, is carried out for a specific purpose, and is marked by lack of sympathy and cold-heartedness. The second one is spontaneous and characterized by loss of control or an acute emotional reaction to provocation. The former type of aggression has been referred to as instrumental, premeditated, proactive, and predatory; the latter has been identified as impulsive, reactive, hostile, emotional, and affective [23]. Distinguishing a reactive aggressive behaviour from a proactive aggressive behaviour is an important first step to better understand their nature and functional value. The usefulness of this two-factor model is also related to the unique capacity of each dimension (i.e., the residual variance) to generate a distinct and theoretically consistent pattern of predictions. The nomological networks of reactive aggression and proactive aggression have been documented in previous studies. For instance, Dodge and Coie [24] found that reactive aggression (either observed or rated by a teacher) is associated with a hostile attributional bias when interpreting the intentions of a peer, whereas proactive aggression is not. A subsequent study revealed that proactive aggression is associated with a positive evaluation of aggression and its consequences, especially in the context of a conflict with peer; this is not true, instead, in the case of reactive aggression [22]. However, an important issue is the capacity to reliably distinguish individuals along these two dimensions. The study of reactive/proactive aggression has risen out of social cognitive theories, such as the frustration-aggression model and social learning theory; the former has been used to describe the provoked emotional outburst associated with reactive aggression, while the latter captures the instrumental function, or positively reinforcing nature, of proactive aggression [18]. Even though reactive aggression and proactive aggression are substantially correlated, the distinction between these two behavioural tendencies appears useful for a qualified study of the processes underlying the display of aggressive conducts, as well as for the study of their different impact on subjects' social adjustment [25]. Furthermore, gender differences in the correlates of proactive and reactive aggression [26], including long-term outcome [27], have been reported, and consequently current findings from male samples may or may not be generalized to females. Excessive dependency in intimate relationships may be related to the development of psychopathology and feelings of possessiveness and intense need for the partner [28]. Henderson et al. [29] noted that the emotional dependency is a risk factor in both sexes, but it manifests itself in different ways: emotionally dependent women are more likely to resort to hostilities and to withdraw emotional support, while men are more likely to experience jealously and control behaviors. A recent research [30] showed that gender and dependency interact to predict guilt (i.e., in conflict situations, dependency is associated with guilt in women, but not in men); it also evidenced that the relationship between dependency and loyalty is mediated by guilt in women, but not in men. Results demonstrated that intimate conflictive situations might elicit different emotional reactions in men and in women. Furthermore, dependency and emotional reactions to conflicts are both predictors of which strategies for managing problems the members of the couple may act in the relationship. It is also important to remark that, even though women feel angry, this anger is not connected to the intention to be aggressive in intimate partner conflicts, as it is among men. The fact that women generally experience guilt more intensely than men has been widely reported too [31, 32]. Kempes et al. [33] have also argued that differences in age in the various study populations could in part be responsible for discrepancy in findings.

The primary objective of this paper is to investigate the levels of correlation between affective dependency and reactive/proactive aggression in a sample of Italian subjects; secondary objectives include an analysis of the distributions obtained considering differences of gender, age, and area of residence, as well as an evaluation of the impact of past intimate relationships on the subject's present attitude towards the bond.

## 2. Methods

### 2.1. Participants

Participants in this study were 3375. 1667 were males (49.7%) and 1698 were females (50.3%); the age ranged from 18 to 52 years; mean = 28.64; SD = 8.99; variance: 80.94, skewness: 0.97 (S.E. = 0.42), and kurtosis: −0.75 (S.E. = 0.84). The sample presented an average age of 28.20 for women (SD = 9.10) and 29.10 for men (SD = 8.87). The selection of the subjects took place through a simple randomization; the only binding condition was that the subject had had at least one sentimental relationship. The total sample covered all major provinces in Central and Southern Italy.

### 2.2. Instruments and Procedure

The following instruments were administered: the* Spouse-Specific Dependency Scale* [3] and the* Reactive Proactive Questionnaire* [34]. The former is a self-reported dependency scale, specific to the primary relationship. It aims at evaluating anxious attachment, exclusive dependency, and emotional dependency as components of the construct of interpersonal dependence in the couple. It consists of three subscales (10 items each) for both men and for women, and it requires approximately 8 minutes to be completed. The question format is a four-level Likert item, from 1 (strongly disagree) to 4 (strongly agree). The three subscales are (1) anxious attachment, (2) exclusive dependency, and (3) emotional dependency. Anxious attachment is identified by separation anxiety, feelings of abandonment by the partner, concern for the feelings of the other person and his/hers whereabouts (“where are you?”, “what are you doing?”), and sensitivity to signs of loss of love and abandonment [31]. This attachment style is mostly associated with the development of psychopathology, such as mood disorders, social anxiety, and depressive symptoms [35]. Exclusive dependence refers to a person relying exclusively on the other member of the couple as fellow and confident, excluding other important relationships, social supports, interests, or activities [36]. Finally, emotional dependency is related to a need for protection and support and a strong trust in the relationship, perceived as essential to self-esteem, identity, and overall functioning of the person [37]. The second administered instrument is the* Reactive Proactive Questionnaire* (RPQ); it is a 23-item measure that yields continuous subscale scores for the reactive (11 items) and proactive (12 items) subscales by summing up the responses. The instructions for the measurements facilitate a nondefensive response and the items tap into the motivational and situational context for the actions. The utility of the RPQ is that it represents a self-report of aggressiveness, in which the person is asked about the reasons of his/her aggressive behaviour and refers to this behaviour in general. Participants indicate how frequently they have experienced each of the items from 0 = never to 2 = often.

Participants in the study were asked to complete both questionnaires thinking about their current intimate relationship or a past one. Among the information requested, they were also asked to indicate the time elapsed since their last affair ended and which of the two partners had decided to end the relationship. The aim of collecting this information was to assess whether the presence of a close or distant previous intimate relationship could influence the perception of the level of dependency and aggression in a new relationship. Furthermore, it was intended to determine whether the voluntary or suffered interruption of a loving relationship was correlated to a greater or lesser inclination to aggression and emotional dependency. Participants were informed that their responses were completely anonymous, and they were guaranteed of absolute confidentiality in the handling of personal data.

### 2.3. Statistical Analysis

All statistical analyses were performed using the Statistical Package for the Social Sciences version 20.0 (SPSS). A *P* value of 0.05 was deemed statistically significant. Data were studied using usual exploratory techniques. Associations of total as well as different types of aggression and dependency scores with different sociodemographic parameters were analysed. Chi square test of significance was performed to find out the association. Pearson's correlation coefficient was used to verify the correlations between the two administered instruments.

## 3. Results

### 3.1. Correlation between Affective Dependence and Aggression

In the whole sample, a positive correlation between emotional dependency and proactive aggression was found (*r* = 0.14, *P* < 0.01). We also proceeded to test correlations between SSDS and RPQ subscales according to gender. In the male sample, we registered a robust correlation between proactive aggression and total aggression (*r* = 0.88, *P* < 0.01), as well as between reactive aggression and total aggression (*r* = 0.91, *P* < 0.01), and between proactive aggression and reactive aggression (*r* = 0.60, *P* < 0.01), while *r* was discreet between anxious attachment and emotional dependency (*r* = 0.52, *P* < 0.01). A slight negative correlation was found between anxious attachment and exclusive dependency (*r* = −0.10, *P* < 0.01). In the female sample, we noted a significant correlation between proactive and reactive aggression (*r* = 0.52, *P* < 0.01) and robust correlations between reactive aggression and total aggression (*r* = 0.91, *P* < 0.01), as well as between proactive aggression and total aggression (*r* = 0.82, *P* < 0.01). We could also evidence a slight correlation between proactive aggression and emotive dependency (*r* = 0.12, *P* < 0.01). With regard to the other measures of dependence, emotional and exclusive dependency presented *r* = 0.22, and emotional dependency with anxious attachment *r* = 0.46; finally, exclusive dependency and anxious attachment showed *r* = 0.33, even with *P* < 0.01.

### 3.2. Affective Dependence with Reference to Gender, Age, and Territory

The sample was articulated into five age classes: 18–24; 25–31; 32–38; 39–45; 46–52. Each class included an interval of seven years, for both homogeneity in the distribution and with the intention to collect the subjects in clusters corresponding to significant stages of the life cycle: (1) completion of the course of study or completion of maturation; shaping of personal identity; (2) job search, apprenticeships, experiences of mobility; (3) work and affective consolidation, parenthood; (4) definition of professional and institutional roles, consolidation of experiences and responsibility; (5) early existential evaluations, enjoyment of achievements, possible regrets. As indicated in Table 1, crosses between affective dependency subscales of SSDS and gender revealed a mean score of 3.82 (SD: 0.65; E.S. 0.01) for males in the emotional dependency subscale. This value was higher than females' mean score, which was 3.44 (SD: 0.54; E.S. 0.01). Exclusive dependency showed similar means for both sexes, with slightly higher values in the women group: 3.66 (SD: 0.59; S.E. 0.01), while 3.51 (SD: 0.47 and S.E. 0.01) was the mean for men. Moreover, both males and females had almost similar mean scores for anxious attachment: 3.62 (SD: 0.89; S.E. 0.02) and 3.58 (SD: 0.84; S.E. 0.01).

With reference to age, as mentioned above the sample was divided into five main classes. Considering this variable, differences of means between classes appear worthy of attention. As reported in Table 2, in the male sample emotional dependency presented the lowest value in the first class (18–24) but reached a very high value, significantly above the average mean (3.87), in the fourth class (39–45). Women showed the lowest emotional dependency in the fifth class (3.37), while the highest value (3.46) was evidenced in the third class of age (32–38). However, mean values in this subscale were lower than men's average.

With regard to the subscale of exclusive dependence, the average values for males were lower than those scored by females, and in the third age class we found the lowest average score (3.47). On the contrary, women showed significantly higher values, rising from the third to the fifth age class (3.77; 3.79; 3.83, resp.). In the second age class the average score was much lower (3.59) and abruptly rose in the following group (3.77). As for the anxious attachment subscale, males had higher values in the second (25–31) and in the last two age groups, in which the average arose considerably (3.72; 3.83). Women scored the highest value (3.68) in the third age group, but it was anyhow lower than the maximum score of man in the same subscale.

Considering the geographic distribution, the sample was divided into two main groups comprising the Central and Southern Italy. It was noted that, with regard to men in the subscale of emotional dependency, there was a scoring difference related to the geographic area of provenance. The value of average male emotional dependency was significantly higher in Southern Italy (3.88) in comparison with Central Italy (3.75); the ANOVA confirmed the significance, with *P* = 0.000 (*P* < 0.01) and *F* of Fisher = 7.9. Anxious attachment was instead only close to significance, with *P* = 0.02 and *F* of Fisher = 6.41, and Eta of, respectively, 0.97 and 0.87 (see Table 3).

In the female sample, differences with respect to geographic area were notable in the subscales of exclusive dependence and anxious attachment. Average values for women were significantly higher in the South (mean of exclusive dependency: 3.72; mean of anxious attachment: 3.63) than in the Centre of the country (mean of exclusive dependency: 3.60; mean of anxious attachment: 3.54); for both associations, ANOVA confirmed the significance with *P* : 0.000 and 0.01 (for *P* < 0.01), *F*: 10.09 and 4.26.

Among the information requested from the subjects, there was also an indication of whether the person had a current relationship or used to have one in the past. We crossed this variable with the subscales of affective dependency. Differentiating by gender, the analysis showed that, in men who had intimate relationships only in the past, the value of exclusive dependency increased (3.57 compared to 3.49), while there was much less anxious attachment (3.38 compared to 3.70). The value of emotional dependency had no relevance in the results. ANOVA confirmed the significance of this association, with *P* = 0.001 and 0.000; *F* = 10.93 and 43.71. Women with an intimate relationship only in the past showed instead a decrease in both exclusive dependency and anxious attachment. The first varied from 3.75 to 3.39, while the second from 3.61 to 3.48. Also in these cases there was significance in the association: (*P* = 0.000 and *F* = 126.62; *P* = 0.002 and *F* = 9.89). Similarly to males, the value of emotional dependency had no impact on the results.

### 3.3. Impact of Last Intimate Relationship on Dependence Attitude

Information on how much time had elapsed since the last relationship for those subjects who did not have a stable love relationship at the time of assessment was among the data requested. We proceeded to cross this variable, distinguishing among people whose latest relationship dated back to less than two years, people whose latest relationship dated back to less than four years, and people whose last relationship dated back to more than four years. Results showed that in males whose latest relationship dated back to more than four years there was an increase in anxious dependency (3.72) and the association revealed significance (*P* = 0.000 and *F* = 3.80). For the emotional dependency there was a slight rise too (from 3.78 to 3.84), but this did not result to be significant (*P* = 0.06). We also crossed dependency subscales, the period of the last relationship, and if the subject had left or had been left by the partner in the past. Results revealed that, in those subjects who had been left by the partner more than four years ago and did not have a new relationship, anxious attachment increased up to 3.79; the relation was significant, with *P* = 0.006; *F* = 1.89. Among females whose latest relationship dated back to more than four years ago, exclusive dependency increased (3.75), and the association revealed significance (*P* = 0.000 and *F* = 13.49). In women who had been left by their partner more than four years ago and were not in a new relationship, exclusive dependency increased instead on average up to 3.80.

### 3.4. Aggression with Reference to Gender, Age, and Territory

Crosses between the two aggression subscales (reactive and proactive), total mean value of RPQ, and gender revealed a reactive aggression mean score for males of 0.86 (SD: 0.42; E.S. 0.01); mean score in proactive aggression subscale was 0.34 (SD: 0.36; E.S. 0.08); mean score for total aggression was 0.60 (SD: 0.35; E.S. 0.08). These values differ from females mean scores, which were 0.77 (SD: 0.36; E.S. 0.08) for reactive aggression, 0.20 (SD: 0.25; E.S. 0.06) for proactive aggression, and 0.49 (SD: 0.27; E.S. 0.06) for total aggression. Considering the mean scores for aggression among age classes, men appeared to present the highest values in the first age class (18–24) for both reactive and proactive aggression subscales and also for total aggression, with values that were, respectively, 0.92, 0.37, and 0.64. In the second class (25–31) we noted a decrease in values, which rose then up again in the third class (32–38), with mean scores 0.84, 0.34, and 0.59. In the last two age classes values tended to decrease. The association between age group and aggression in men had significance in all of the three measures, with *P* = 0.000 and *F* = 6.28 for the reactive subscale; *P* = 0.01 and *F* = 3.30 for the proactive subscale; *P* = 0.000 and *F* = 5.51 for the total aggression measure. Among women, aggression values tended to be higher in the first and second age range (18–24, 25–31) and then fell significantly. More in detail, the three values for reactive aggression, proactive aggression, and total aggression in the first age class were 0.82, 0.22, and 0.52, respectively, while for the second class they were 0.77, 0.20, and 0.49. The association between age group and aggression in women had significance in all three measures, with *P* = 0.000 and *F* = 10.74 for the reactive subscale; *P* = 0.01 and *F* = 4.79 for the proactive subscale; *P* = 0.000 and *F* = 10.32 for the total aggression measure. Considering the geographic distribution (Centre and South of Italy), we could note differences among the values scored by males in the third and fifth age class (32–38 and 46–52), residing in the Centre and in the South of the country. For the third age group, reactive aggression values ranged from 0.78 (Centre) to 0.88 (South); for proactive aggression they were 0.29 (Centre) and 0.36 (South); finally, for total aggression values were 0.54 in the Centre and 0.62 in the South. The oscillation in the fifth class (46–52) was even more meaningful: reactive aggression scores were 0.73 (Centre) and 0.84 (South); proactive aggression scores were 0.18 (Centre) and 0.29 (South), and total aggression values ranged from 0.45 (Centre) to 0.56 (South). The association between territory of residence and aggression for men showed significance in all the three measures, with *P* = 0.000 and *F* = 6.28 for the reactive subscale; *P* = 0.01 and *F* = 3.30 for the proactive subscale; *P* = 0.000 and *F* = 5.51 for the total aggression measure. We could note differences worthy of attention among women as well: similarly to men, in the second age class we registered a higher value of reactive aggression in southern residents (0.82) in comparison with residents in Central Italy (0.72); from the third age group (32–38), instead, the trend was reversed: females from the Centre had higher values for reactive aggression (0.73 versus 0.67) than for proactive aggression (0.20 versus 0.11), while for total aggression the score was 0.47 (versus 0.39). In the fourth and fifth age class, the direction of the values was confirmed, but their amplitude was less pronounced. The association between territory of residence and aggression in the female group had significance in all the three measures, with *P* = 0.000 and *F* = 10.74 for the reactive subscale; *P* = 0.01 and *F* = 4.79 for the proactive subscale; *P* = 0.000 and *F* = 10.32 for the total aggression measure. The fact that subjects were or were not involved in a relationship did not influence the distribution of scores for the measure of aggression; the association was therefore not significant. Considering distribution by marital status, men reported higher levels in reactive, proactive, and total aggression measures in the groups of widowed (1.31; 0.65; 0.98) and divorced (1.07; 0.57; 0.82), and the association was significant, with *P* = 0.001; *F* = 2.82 for reactive aggression associated with marital status; *P* = 0.003; *F* = 3.32 for proactive aggression associated with marital status; *P* = 0.002; *F* = 3.56 for total aggression associated with marital status. Women revealed higher values for reactive and total aggression (0.87 and 0.54) among the group of the separated, while they reported the lowest values for reactive and total aggression (0.58 and 0.34) among the divorced group.

## 4. Discussion

The link between emotional needs of individuals and aggressive behaviour was confirmed by the results of the study, with particular emphasis on the relationship between emotional dependency and proactive aggression. Despite being slight, the result is worthy of attention because it allows us to corroborate the hypothesis of the fundamental distinction between reactive and proactive aggression, anchoring the latter more strictly to emotional dependency. We know that among the three constructs that compose dependence (exclusive dependency, attachment anxiety, and emotional dependency), the last one refers to basic need for protection and support and is expressed as a need of intimate relationship as essential to self-esteem, identity, and overall functioning of the person. It is therefore understandable that this need for long-term preservation of the subject's intimate relationship may be more associated with strategy and not merely reactive aggressive behaviour. The correlation may also be attributed to the fact that the sample was composed of a nonclinical population, which declared to have low levels of aggressiveness. Aggression of anxious and exclusively dependent subjects need a real and present threat to manifest and are generally reactively channelled; it is therefore understandable that the conditions of absolute normality in which the administration of the instruments occurred did not solicit these levels of aggressive behaviour in the self-report registration. The results showed that the correlation between proactive aggression and emotional dependence tends to occur regardless of contextual situations and time, to involve personality dimensions, and to express a strategic orientation to the active control of the relationship in order to pursue the subject's objectives/needs. For a better understanding of aggressive behaviour within the couple, it is essential to assess the evolution and specificity of emotional needs in relation to the individual's life cycle, taking into account the gender, the sociocultural influences that affect the couple, and the structuring of harmony/disharmonies.

Emotional dependency in men tends to grow throughout life, but it reaches its maximum between the ages of 39 and 45. For women, the trend in this scale is irregular: maximum levels of emotional dependency are reached around 32 and 38 years, and then they decrease to get to the minimum level in subjects over 45. With regard to exclusive dependency, the values are especially low in males aged 32 to 38 years, while women have a continued growth in these values from the age of 32 until the maturity of the fifties. In the age class 25–31 there is a high level of anxious attachment in males, which then decreases, but rises again in the last two age groups. In women, anxious attachment is mostly evidenced in subjects aged 32 to 38 years. It is clear that these differences affect the management of personal relationships in the couple, because the different personal needs and requests are often not reconcilable and can be the basis for increasing strain. Similarly, it is evident that the pressure of sociocultural context with the structuring of attachment styles helps to determine the scope, frequency, and intensity of the demands made to the partner, as well as feeding fears of loss, abandonment, or betrayal. According to Bornstein [38], emotional dependency refers to a need for protection and support and a strong confidence in the relationship as an essential element for self-esteem, identity, and overall functioning of the person. Therefore, it is not surprising what emerged about men who, at a critical age (39–45), are faced with significant challenges inside and outside the domestic environment, in the maintenance, construction, or recovery of economic and social conditions, especially in times of rapid changes, crises, and internal and external pressure towards the couple. It is natural to think that, facing increasing pressure and stress induced from the outside, males are directed defensively to the partner in order to obtain an anchor when confronting with the fear of loss and to gain support for self-esteem, narcissistic confirmation, and emotional regulation. Women in the age group between 32 and 38 years are called to address the simultaneous tasks of consolidation and affective labour, taking charge of parenting in a delicate moment of perception of physical and psychological changes. In this phase there is the highest demand for emotional closeness and emotional support. The fear of not succeeding in achieving goals, not only material, translates into an amplification of requests for fusion, with an increase of expectations and often frustrations when confronted with reality. These requests are accompanied by a parallel trend (which extends up to 50 years old) to develop behaviors configured as exclusive dependence. The woman in the couple confides exclusively in a companion-confident, with the exclusion of other important relationships and other sources of social support, interests, or activities. Men in the 32−38 years range, instead, record the lowest value in this inclination, because this life phase usually coincides with the consolidation of roles and functions, which does not exclude but opens and includes external networks to the subject. It is evident that in some cases the misalignment of this dimension is the reason for strong tensions and frustrations of mutual expectations. For men, the fears of loss and abandonment are more evident in the last two age groups. Fears of separation and feelings of abandonment are at the base of the anxious attachment, which manifests through a more open concern for the feelings of the partner, the desire to constantly know her intentions, jealousy, and marked sensitivity to signs of loss of affection.

The comparison between geographical areas has shown that higher values of emotional dependency in men and higher values of exclusive dependence and attachment anxiety in women are recorded in Southern Italy. These data may well reflect the cultural and social influences on the relationship. Cultural heritage that characterizes the context in which a person lives is a delicate element to identify the roots and the pervasiveness of certain behaviors in the intimate and personal sphere, the degree of acceptability of the same, and possibly resistance to change. Couples and families nowadays belong to or are members of different cultural contexts at the same time, which means not only that they are assigned to a group or physical context but also that they have different cultural contexts in the areas of sexuality, food, rituals, concept of gender, relevance attached to births, deaths, consumption, and links.

The study also examined the attitude of those who are not currently in a relationship but have had one in the past. An interesting result highlighted that if the previous relationship dated back to more than four years, in men we observe a trend towards higher values of anxious attachment, whereas in women there is an increase of exclusive attachment. For both genders, these values rise if the affair was interrupted by the will of the other member of the couple. This result emphasizes in men the fear of not being able to keep a new bond; the interruption of the previous relationship triggered fears and insecurities that are likely to have convinced the subjects to stay single for a long period, and if he were to meet a new partner, the relationship would be strongly influenced by this attitude. In women, the interruption of the past bond activates an attitude of exclusive dependency, therefore producing pressing demands of exclusivity towards the potential partner and a preference for happy isolation. With reference to marital status of men, anxious attachment is also significant in the divorced and widowed group, in which of course the experience of separation and overt loss (divorce and bereavement) have triggered fears and insecurities that shaped the attitude of these subjects against current romantic relationships.

With regard to the distribution of the values of aggression by age, it is significant to note that in men the higher levels are concentrated in the 18 to 24 and in the 32 to 38 years group. In the first case, we can consider a greater propensity to impulsivity and less control of the reactions. The second group identifies instead the phase in which subjects face the transition to adult life and the new responsibilities may produce rising levels of aggression. For women, it is possible to note that the values tend to decrease from the second age group and basically remain low until the 52 years limit.

Performing comparison by geographical area and by sexes other data emerge. In males, the third and fifth age classes (32–38; 46–52) are important in the south of Italy, where residents show more pronounced values in reactive aggression, proactive aggression, and in the total score of aggression. The specificity of the high values in the southern 46–52 years group recalls the impact of cultural and social factors: a mature man is required to show an appropriate level of assertiveness and energy in his relations, in order to exhibit a tangible expression of his will. Thus, this may be a culturally coded mode of communication, counterbalanced by the more accommodating attitude of the women, at least from a certain age. In fact, for men we found a more pronounced reactive aggression value in the South for the first two age ranges, while from the third to the fifth groups there was a reversal of this tendency. A final remark on the relationship between marital status and aggressiveness is that among men there was a high level of aggression in the widowed and divorced group, while for women there was an increase in total and reactive aggression among the group of the separated, while the lowest values were among the divorced. Clearly, the experience of loss and final dissolution of a relationship involves a more difficult elaboration for men, who seem to harbour a very strong brooding resentment, ready to discharge the accumulated tension. The loss of status and the limitation of material resources often borne by men following a divorce or bereavement are also to be considered. Among the most significant values is the sharp rise in proactive aggression, which would lead us to believe that aggression becomes a stable mode oriented to the achievement of manipulative purposes, regardless of the perception of threat solicited by others. In women it is often the exact time of separation that undermines the exclusivity of the relationship and activates the major anxieties of loss. Being it often a condition of suspension and hybrid transition to new settlements that have not yet been defined, this activates in women a considerable aggression as a response to injury, but above all as intolerance of uncertainty. From this point of view, divorce represents a defined state and, though severe, better accepted.

## 5. Conclusions 

Results underline a positive correlation between emotional dependency and proactive aggression. Despite the slight rate, this is worthy of attention because it allows us to corroborate the hypothesis of a fundamental distinction between reactive and proactive aggression, anchoring the latter more strictly to emotional dependency. Unlike anxious attachment and exclusive dependency, the correlation between proactive aggression and emotional dependency tends to occur regardless of contextual situations and time, as well as to involve the deep dimensions of personality, and it expresses a strategic orientation to the active control of the relationship to pursue personal objectives/needs. Emotional and relational needs emerge differently in different life ages and often do not coincide in the couple. This lack of understanding and the misalignment may be an element that contributes to the emergence of aggressive phenomena. The observations emerged in terms of territorial distinctions highlight the need to evaluate the role of social and cultural components in shaping the *t* regulation of behaviour and emotional expression. The study results emphasize the difficulty for males to overcome situations of separation and loss, such as bereavement and divorce, whose effects are much stronger if they occurred in the distant past of the subject and may lead to rising levels of anxious attachment and a significant increase in aggression in both the reactive and proactive component. In women we observed a peculiar sensitivity in the phase of separation from a previous relationship, which is generally accompanied by an increase in the exclusive and totalizing requests addressed to the bond and which may further contribute to exacerbate situations of tension. The time interval since the last intimate relationship may also differentially affect the levels of dependency and type of aggression expressed by the subjects. It would certainly be desirable to continue the study with an analysis of the levels of codependence and influence of sociocultural factors in the manifestation of reactive and proactive aggression in a sample of couples.

## Figures and Tables

**Table 1:** Affective dependency means and gender.

	Mean	Std deviation	*N*
Total aggression males	.6024	.35480	1677
Reactive aggression males	.8630	.42409	1677
Proactive aggression males	.3417	.36652	1677
Emotional dependency males	3.8200	.65818	1677
Exclusive dependency males	3.5162	.47157	1677
Anxious attachment males	3.6210	.89973	1677

Total aggression females	.4903	.27433	1698
Reactive aggression females	.7778	.36977	1698
Proactive aggression females	.2028	.25459	1698
Emotional dependency females	3.4407	.54038	1697
Exclusive dependency females	3.5837	.77830	1698
Anxious attachment females	3.6640	.59732	1697

**Table 2:** Dependence and aggression among class of age.

Class of age		Emotional dependency	Exclusive dependency	Anxious attachment	Proactive aggression	Reactive aggression	Total aggression
1.00	Mean	3.5947	3.5789	3.5669	.2906	.8712	.5809
	*N*	1472	1472	1472	1472	1472	1472
	Std deviation	.61366	.54587	.79457	.31434	.40937	.32407

2.00	Mean	3.6607	3.5535	3.5992	.2697	.8037	.5367
	*N*	908	908	908	908	908	908
	Std deviation	.60929	.51284	.85222	.32468	.38805	.31238

3.00	Mean	3.6640	3.6034	3.6336	.2638	.7858	.5248
	*N*	410	410	410	410	410	410
	Std deviation	.63585	.53448	.87377	.34167	.40081	.33262

4.00	Mean	3.6767	3.6552	3.6751	.2570	.7521	.5046
	*N*	327	327	327	327	327	327
	Std deviation	.69781	.57465	.89229	.36122	.38225	.33137

5.00	Mean	3.6002	3.6853	3.6724	.2033	.7272	.4653
	*N*	257	257	257	257	257	257
	Std deviation	.69527	.59193	.92822	.26686	.36822	.28538

Total	Mean	3.6293	3.5905	3.6023	.2718	.8201	.4460
	*N*	3374	3374	3374	3374	3374	3374
	Std deviation	.6309	.54347	.84091	.32274	.39991	.32175

Sig.		.035	.001	.115	.001	.000	.000

*F*		2.583	4.413	1.859	4.412	13.165	10.490

Mean square		1.026	1.298	1.313	.458	2.075	1.074

df		4	4	4	4	4	4

Sum of square		4.105	5.192	5.253	1.831	8.302	4.296

Eta		.055	.072	.047	.072	.124	.111

Eta squared		.003	.005	.002	.005	.015	.012

**Table 3:** Dependence and aggression among geographical areas.

Geographical area		Emotional dependency	Exclusive dependency	Anxious attachment	Proactive aggression	Reactive aggression	Total aggression
Center	Mean	3.5971	3.5526	3.5454	.2857	.8109	.5483
	*N*	1457	1457	1457	1457	1457	1457
	Std deviation	.63177	.52532	.83476	.33736	.41434	.33540

South	Mean	3.6680	3.6255	3.6685	.2585	.8281	.5433
	*N*	1626	1626	1626	1626	1626	1626
	Std deviation	.63981	.55194	.84820	.30815	.38911	.31035

Other	Mean	3.5739	3.5848	3.5170	.2768	.8216	.5492
	*N*	291	291	291	291	291	291
	Std deviation	.56147	.57326	.80417	.32550	.38594	.31542

Total	Mean	3.6293	3.5905	3.6023	.2718	.8201	.5460
	*N*	3375	3375	3375	3375	3375	3375
	Std deviation	.6309	.54347	.84091	.32274	.39991	.32175

Sig.		.002	.001	.000	.063	.492	.897
*F*		6.107	6.972	9.932	2.764	.710	.108
Mean square		2.423	2.052	6.986	.288	114	.011
df		2	2	2	2	2	2
Sum of square		4.847	4.104	13.974	.575	.227	.022
Eta		.060	.064	.077	.040	.021	.008
Eta squared		.004	.004	.006	.002	.000	.000
